# Representativeness of participants in a randomized controlled trial on modalities of monitoring oral HIV pre-exposure prophylaxis use

**DOI:** 10.1186/s12981-026-00886-1

**Published:** 2026-04-13

**Authors:** Marije L. Groot Bruinderink, Maarten F. Schim Van Der Loeff, Florien Dusseldorp, Katja Van Der Velde, Laura Blitz, Jean-Marie Brand, Colette Van Bokhoven, Joey Woudstra, Koenraad Vermey, Sophie Boers, Maudy Sluimer, Hannelore M. Götz, Anders Boyd, Lotte Werner, Maaike L. Soors d’Ancona, Frenk Van Harreveld, Maria Prins, Elske Hoornenborg, Udi Davidovich, Vita W. Jongen

**Affiliations:** 1 Department of Infectious Diseases, Public Health Service Amsterdam, GGD Amsterdam, Nieuwe Achtergracht 100, 1018 WT Amsterdam, The Netherlands; 2 Department of Psychology, University of Amsterdam, Amsterdam, The Netherlands; 3 Department of Internal Medicine, Amsterdam UMC location University of Amsterdam, Meibergdreef 9, Amsterdam, The Netherlands; 4 Amsterdam Institute for Immunology & Infectious Diseases (AII), Amsterdam, the Netherlands; 5 Amsterdam Public Health research institute (APH), Amsterdam, the Netherlands; 6 Department of Sexual Health, Public Health Service of Haaglanden, The Hague, The Netherlands; 7 Department of Sexual Health, Public Health Service of Gelderland-Zuid, Nijmegen, The Netherlands; 8 STI AIDS, Amsterdam, The Netherlands; 9 Department of Infectious Diseases, Public Health Service Rotterdam Rijnmond, Rotterdam, The Netherlands; 10 Stichting HIV Monitoring, Amsterdam, the Netherlands; 11 Public and Occupational Health, Amsterdam UMC Location University of Amsterdam, Meibergdreef 9, Amsterdam, The Netherlands; 12 Quality of Care, Amsterdam Public Health Research Institute, Amsterdam, The Netherlands; 13 Amsterdam UMC, Centre for Sustainable Healthcare, Amsterdam, The Netherlands

**Keywords:** Pre-exposure prophylaxis, HIV prevention, MSM, Transgender and gender diverse persons, Sexually transmitted infections

## Abstract

**Background:**

We assessed whether participants in the EZI-PrEP study, a non-inferiority trial evaluating online and six-monthly monitoring of oral HIV pre-exposure prophylaxis (PrEP), represent the broader population of PrEP users in the Netherlands.

**Methods:**

We conducted a cross-sectional study using routinely collected data from September 2021 to August 2022 at four centres for sexual health (CSHs) in the Netherlands. Socio-demographic characteristics, sexual behaviour, and prevalence of bacterial STIs were compared between EZI-PrEP participants at baseline and other PrEP users during their first PrEP monitoring visit during the same period.

**Results:**

The analysis included 469 EZI-PrEP participants and 5161 other PrEP users, of whom 99% and 96% were men who have sex with men (MSM), respectively. EZI-PrEP participants were less often transgender or gender-divers persons (TGDP) (1% vs. 4%, *p* < 0.001), older (median age = 36 vs. 34 years, *p* = 0.004), more often born in the Netherlands (68% vs. 58%, *p* < 0.001), and more often completed a university/college degree (81% vs. 76%, *p* = 0.01). They also reported more group sex (38% vs. 33%, *p* = 0.023) and condomless anal sex (95% vs. 92%, *p* = 0.004), but less often sex work (1% vs. 6%, *p* < 0.001). Prevalence of bacterial STIs were no different between groups (19% vs. 18%, *p* = 0.77).

**Conclusions:**

The comparable STI prevalence suggests no difference in risk for HIV acquisition among EZI-PrEP participants and other PrEP users, making study outcomes applicable to a broader population of PrEP users. However, under-representation of TGDPs, sex workers, individuals not born in the Netherlands, and individuals without university or college degree may limit generalizability.

**Supplementary Information:**

The online version contains supplementary material available at 10.1186/s12981-026-00886-1.

## Introduction

 Oral HIV pre-exposure prophylaxis (PrEP) is highly effective against HIV acquisition and is an important tool in curbing the HIV epidemic [1–5]. However, uptake and retention in PrEP programs is suboptimal [6–9]. Reported barriers to in-clinic PrEP care include anticipating or experiencing stigma, discrimination and lack of privacy at the PrEP providing facility, having difficulties with taking time off from work or other obligations to attend PrEP monitoring visits, travel costs [9–17], and limited capacity of PrEP programmes due to staff shortages or budget limitations [18, 19]. Some of these barriers could potentially be mitigated by reducing the frequency of in-clinic PrEP monitoring visits, for example, through online PrEP monitoring or less frequent in-clinic visits. While both approaches have been implemented in various formats and settings worldwide, their effect on PrEP adherence among PrEP users overall remains understudied.

The EZI-PrEP study was initiated to assess whether reducing the number of in-clinic PrEP monitoring visits would affect PrEP adherence. Designed as a 2 × 2 factorial, non-inferiority, randomized-controlled trial (RCT), the study aimed to determine, in terms of prevention-effective adherence, whether 6-monthly and online PrEP monitoring are non-inferior to 3-monthly and in-clinic PrEP monitoring [20]. The randomized nature of the EZI-PrEP study allows to control for differences in both measured and unmeasured characteristics between study arms and as such, enables to establish a causal relationship between the modalities and frequencies of PrEP monitoring and adherence. Moreover, RCTs are known to commonly have selective eligibility criteria and stringent protocols, which are helpful in reducing statistical noise and ensuring appropriate assessment of both exposures and study outcomes (i.e., internal validity) [21–23]. However, applying selective criteria and stringent protocols might come at the cost of studying populations that are not representative of the broader target population (i.e., reducing generalizability) [21, 22]. Assessments of potential issues in generalizability are rarely conducted for RCTs, since data on the target population are often lacking.

The EZI-PrEP study was nested within the much larger, government-funded National PrEP pilot Programme (NPP), which offers PrEP care to persons at increased risk for HIV acquisition [24]. As part of this programme, data on demographics, sexual health and sexually transmitted infections (STIs) are routinely collected. Assuming that PrEP users of the NPP closely represent populations prioritized for PrEP care, comparing data between EZI-PrEP and the NPP could allow assessment of potential issues in generalizability of the EZI-PrEP trial.

The aim of the current study was therefore to compare the distributions of socio-demographic characteristics, sexual behaviour, and prevalence of bacterial STIs of EZI-PrEP study participants to other PrEP users, not enrolled in the study, who were seeking PrEP care at the same sites. A secondary objective was to assess variation in participant characteristics between the four study sites.

## Methods

### Study setting

The EZI-PrEP study was embedded in the government-funded Dutch National PrEP pilot Programme (NPP) [24]. The NPP operated between 1 July 2019 and 31 July 2024 and was the most common route to receive PrEP care in the Netherlands. The programme offered free-of-charge PrEP care and subsidized PrEP tablets to persons at increased risk for HIV acquisition [24]. The NPP was implemented by 23 centres for sexual health (CSH) of public health services in the Netherlands. The NPP was capped at 8,500 PrEP users; for each centre for sexual health a maximum number of PrEP users was set. At the start of the EZI-PrEP study, waiting lists for NPP enrolment were in place.

The EZI-PrEP study was conducted at four centres for sexual health (CSH) located in Amsterdam, Rotterdam, The Hague and Nijmegen. These centres are among the largest centres for sexual health in the Netherlands; together accounting for approximately 40% of the NPP’s total capacity, participated in the EZI-PrEP study. Amsterdam, Rotterdam and The Hague are considered large cities in the Netherlands (i.e., between 555,000 and 890,000 inhabitants per 1 January 2022), while Nijmegen is a medium-sized city (approximately 177,300 inhabitants per 1 January 2022) located in the eastern part of the country. These centres serve PrEP users in their respective public health service catchment areas which includes surrounding smaller municipalities.

### Design, procedures and population

We conducted a cross-sectional analysis using routinely collected data on socio-demographics, sexual behaviour, PrEP use, and the testing and diagnosis of bacterial STIs. These data were derived from PrEP initiation and monitoring consultations following routine procedures of the NPP. PrEP consultations include tailored counselling on PrEP use and sexual health, using motivational interviewing techniques. Each PrEP consultation also included screening for HIV and bacterial STIs (i.e., *Chlamydia trachomatis*, *Neisseria gonorrhoeae*, and *Treponema pallidum*). Testing for *Chlamydia trachomatis* and *Neisseria gonorrhoeae* was conducted at three anatomical sites (rectal, urogenital, and pharyngeal). Participants diagnosed with an STI were offered free-of-charge treatment in accordance with standard PrEP care protocols. A pre-defined selection of routinely collected data is pseudonymized and sent to the National STI surveillance database of the National Institute for Public Health and the Environment (RIVM, Bilthoven, the Netherlands) for monitoring and surveillance purposes. Individuals participating in the NPP may opt-out of sharing their data with this national STI surveillance database. After a PrEP consultation, individuals could obtain PrEP; they were given the number of tablets needed until their next consultation, based on their planned dosing schedule (i.e., daily or intermittent).

In this analysis we defined two groups of PrEP users: EZI-PrEP participants and other PrEP users. EZI-PrEP participants were all individuals who were enrolled in the NPP and in the EZI-PrEP study. Other PrEP users were individuals who were enrolled in the NPP in one of the four CSHs participating in EZI-PrEP, did not opt-out of the use of their data, and did not participate in the EZI-PrEP study. Individuals eligible for PrEP care via the NPP were men who have sex with men and transgender or gender-diverse persons who reported condomless anal sex with a male partner with unknown or detectable viral load, had a rectal STI, or had used HIV post-exposure prophylaxis (PEP) in the past 6 months [25].

The design and procedures of the EZI-PrEP study have been described previously [20]. In brief, participants were recruited by trained nurses during regular PrEP monitoring visits and from the waiting lists for enrolment in the NPP, at one of the four participating CSHs of Public Health Services in Amsterdam, Rotterdam, The Hague, and Nijmegen. The number of participants included by each CSH was roughly proportional to each centre’s maximum number, set by the NPP. Eligible individuals: (1) were 18 years or older; (2) lived in the catchment area of a participating CSH; (3) were able to complete informed consent, medical history, a daily diary, and questionnaires in English or Dutch; (4) had an email address; (5) owned a smartphone capable of running the study application (i.e., to access the electronic daily diary and online care elements used throughout the trial); (6) had daily access to an internet connection; and (7) being able to complete online bank transactions (i.e., to pay for PrEP home delivery costs if indicated). Individuals were excluded from participation in the EZI-PrEP study in case of an HIV diagnosis or when, based on assessments by the study nurse or physician, online-mediated or 6-monthly monitoring would be inappropriate due to medical or psychosocial conditions (e.g. severe mental health problems, unstable housing, or anticipated medical treatments unrelated to PrEP care that could interfere with compliance with study procedures) [20]. After the PrEP consultation and explanation of the study, participants provided signed informed consent and were randomized to one of four study arms: (1) in-clinic monitoring every 3 months, (2) in-clinic monitoring every 6 months, (3) online monitoring every 3 months, or (4) online monitoring every 6 months. After enrolment, EZI-PrEP participants were requested to complete an online baseline questionnaire including questions about income level, choice of PrEP regimen, mental well-being through the Patient Health Questionnaire-9 (PHQ-9) [26], sexual compulsivity scale (SCS) [27, 28], Alcohol Use Disorders Identification Test (AUDIT) [29–31], and Drug Use Disorders Identification Test (DUDIT) [32, 33].

In the main analysis comparing EZI-PrEP participants to other PrEP users, we used routinely collected data from the national surveillance database. For EZI-PrEP participants, we used data from the PrEP consultation that was part of their enrolment visit. For other PrEP users, we used data from the first PrEP consultation of each individual between 21 September 2021 and 9 August 2022 (i.e., the inclusion period of the EZI-PrEP trial) (Textbox 1). Women and individuals < 18 years old who were receiving PrEP care at one of the CSHs were excluded from the analyses, as these groups were not eligible for participation in the EZI-PrEP trial. In the secondary analyses comparing EZI-PrEP participants across centres for sexual health, we used the surveillance data and the data from the baseline questionnaire.

**Textbox 1** Overview of the number of participants, start and end date of the inclusion period, per Centre for Sexual Health.

**Table:** 

Centre for Sexual Health	Number of participants	Start date	End date
Amsterdam	252	21 September 2021	29 July 2022
Rotterdam	112	11 February 2022	9 August 2022*
The Hague	75	09 November 2021	29 July 2022
Nijmegen	30	25 January 2022	29 July 2022

*We used surveillance data of other PrEP users from Rotterdam until 29 July 2022

### Variables

The analyses included variables on socio-demographic characteristics (e.g., age, self-identified gender, country of birth and highest completed or current educational level), sexual behaviour in the preceding 6 months (e.g., number of sexual partners, condom use, group sex, sex work, and chemsex) and PrEP use in the preceding 3–6 months. Chemsex was defined as use of crystal methamphetamine, gamma-hydroxybutyrate (GHB)/gamma-butyrolactone (GBL), mephedrone or ketamine around the time of sex [34]. Chlamydia and gonorrhoeae infections were defined as a positive test result in at least one anatomical location; while infectious syphilis was defined as being diagnosed with primary syphilis, secondary syphilis or early latent syphilis.

Only for EZI-PrEP participants, a PHQ-9 score ≥ 5 was used to indicate symptoms of depression [26], and an SCS score ≥ 24 was used to indicate a greater impact of sexual thoughts on daily functioning and an inability to control sexual thoughts or behaviours [27, 28]. Based on the AUDIT scores, we categorized alcohol use as follows: no or low-risk alcohol consumption (0–7), hazardous alcohol consumption (8–15), harmful alcohol consumption (16–19), and likely dependent on alcohol (≥ 20) [29–31, 35]. Based on the DUDIT scores we created three categories: no drug use related problems (≤ 5), harmful drug use (6–24), and likely dependent on drugs (≥ 25) [32, 33].

### Statistical analysis

Characteristics were compared (1) between CSHs overall, and not pairwise, (in only EZI-PrEP participants) and (2) between EZI-PrEP participants and other PrEP users using Wilcoxon rank-sum tests for continuous variables, and Pearson’s χ^2^ or Fisher’s Exact for categorical variables. The comparison between EZI-PrEP participants and other PrEP users was also conducted separately within each centre for sexual health. As all analyses were considered exploratory [36], we did not adjust p-values for multiple comparisons.

All analyses were performed using STATA (v17.0, STATA Corporation, College Station, TX, USA).

## Results

### Baseline characteristics of EZI-PrEP participants

Between 21 September 2021 and 9 August 2022, 469 EZI-PrEP participants were included: 464 identified as male and five as transgender or gender-diverse (Table 1).

**Table 1:** Baseline socio-demographic characteristics and sexual behaviour of EZI-PrEP participants and other PrEP users of the Dutch National PrEP pilot Programme at the Centres for Sexual Health in Amsterdam, Rotterdam, The Hague and Nijmegen, September 2021–August 2022

	EZI-PrEP (*n* = 469)		NPP (*n* = 5161)		*p*-value^2^
	*n* ^1^	%^1^	*n* ^1^	%^1^	
**Centre for Sexual Health**					
Amsterdam	252	54%	3,030	59%	0.003
Rotterdam	112	24%	1,234	24%	
The Hague	75	16%	543	11%	
Nijmegen	30	6%	360	7%	
**Demographic characteristics**					
**Self-identified gender**					
Male	464	99%	4954	96%	0.001
Transgender or gender-diverse	5	1%	207	4%	
**Age in years**					
Median, [IQR]	36	[29–47]	34	[28–44]	0.005
< 25years^3^	48	10%	655	12%	0.046
25–34 years	168	36%	1,975	38%	
35–44 years	111	24%	1,253	24%	
≥ 45 years	142	30%	1,278	25%	
**Country of birth**					
The Netherlands^4^	319	68%	2,982	58%	< 0.001
Western and Central Europe and North America	55	12%	548	11%	
Eastern Europe and Central Asia	14	3%	263	5%	
Middle East and North Africa	11	2%	370	7%	
Latin America and the Caribbean	39	8%	627	12%	
Asia and the Pacific	22	5%	275	5%	
Sub-Saharan Africa	9	2%	83	2%	
Missing/unknown	1		13		
**Highest completed or current education level** ^5^					
None, primary or other	12	3%	321	6%	0.001
Secondary	72	16%	791	17%	
College/university	377	82%	3,562	77%	
Missing/unknown	8		487		
**PrEP use past year**					
No use	54	12%	565	11%	0.402
Used PrEP 4–12 months prior to inclusion	7	1%	126	2%	
Used PrEP in the 3 months before inclusion	408	87%	4,389	86%	
Unknown/missing	0		81		
**Sexual behaviour in preceding 6 months**					
** Number of sex partners**,** median [IQR]**^**6**^	7	[4–15]	6	[3–14]	0.056
** Had insertive anal sex**	406	87%	4,404	85%	0.468
** Had receptive anal sex**	400	85%	4,329	84%	0.426
**Had condomless anal sex**	447	95%	4,726	92%	0.005
** Had condomless insertive anal sex**	394	84%	4,092	79%	0.015
** Had condomless receptive anal sex**	388	83%	4,018	88%	0.014
** Had group sex** ^**7**^	168	38%	1672	33%	0.024
** Did sex work** ^**8**^	3	1%	291	6%	< 0.001
** Had chemsex** ^**9**^	123	26%	1,157	22%	0.060
** Injected drugs during or around sex** ^**10**^	3	1%	55	1%	0.276
**Bacterial STI diagnoses**					
** Any bacterial STI** ^**11**^	84	19%	894	18%	0.766
*** Chlamydia trachomatis*** ^***13***^	40	9%	443	9%	0.973
*** Neisseria gonorrhoea*** ^***12***^	45	10%	524	11%	0.696
** Infectious syphilis** ^**14**^	8	2%	78	2%	0.737

Bold values indicate main categories

Proportions are based on complete data; missing/unknown data excluded from calculations

EZI-PrEP: E-Health for Zero Infections—facilitating access to and use of Pre-Exposure Prophylaxis in the Netherlands; IQR: interquartile range; PrEP: pre-exposure prophylaxis; STI: sexually transmitted infection

1. Unless stated otherwise

2. Based on Wilcoxon rank-sum tests for continuous variables and Pearson’s χ^2^ or Fisher’s exact for categorical variables

3. Binary comparison of 18–25 year old vs. ≥25 years old, p-value 0.123

4. Binary comparison of those born in the Netherlands vs. not born in the Netherlands, p-value < 0.001

5. None or Primary or other: no education, elementary school, lbo, mavo, vmbo, mbo-1; Secondary: mbo-2-4, havo, vwo; University or College: university of applied sciences, university. Binary comparison of college/university vs. secondary, none/primary/other, p-value 0.007

6. Missing/unkown: EZI-PrEP (*n* = 12), NPP (*n* = 90)

7. Missing/unkown: EZI-PrEP (*n* = 31), NPP (*n* = 102)

8. Missing/unkown: EZI-PrEP (*n* = 21), NPP (*n* = 570)

9. Chemsex sex under influence of one or more of the following drugs: crystal methamphetamine; mephedrone; GHB/GBL; ketamine. Missing/unkown: EZI-PrEP (*n* = 1), NPP (*n* = 13)

10. Drugs included: 3-mmc; heroin; ketamine; cocaine. Missing/unkown: EZI-PrEP (*n* = 1), NPP (*n* = 13)

11. Having at least 1 bacterial STI. Missing/unknown: EZI-PrEP (*n* = 15), NPP (*n* = 178)

12. Missing/unknown: EZI-PrEP (*n* = 15), NPP (*n* = 169)

13. Missing/unknown: EZI-PrEP (*n* = 16), NPP (*n* = 171)

14. Infectious syphilis: primary syphilis, secondary syphilis or early latent syphilis. Missing/unkown: EZI-PrEP (*n* = 9), NPP (*n* = 87)

Median age was 36 years [interquartile (IQR) = 29–47], with 48 (10%) EZI-PrEP participants being < 25 years old. Most participants (68%, *n* = 319) were born in the Netherlands, 377 (82%) completed or pursued a university or college degree, and 408 (87%) had used PrEP in the 3 months preceding enrolment. Twelve participants (3%) had no formal education or only a primary education. The median number of sex partners in the 6 months before enrolment was 7 (IQR = 4–15). During the same period, 447 (95%) EZI-PrEP participants reported condomless anal sex, 168 (38%) group sex, 123 (26%) chemsex, and 3 (1%) sex work. 84 (19%) participants were diagnosed with at least one bacterial STI at enrolment: *Neisseria gonorrhoea* infection, *n* = 45 (10%); *Chlamydia trachomatis* infection, *n* = 40 (9%); infectious syphilis, *n* = 8 (2%).

Almost half (*n* = 203, 47%) of EZI-PrEP participants, scored five or higher on PHQ-9, suggesting mild, moderate or severe symptoms of depression. Few EZI-PrEP participants were likely dependent on alcohol (*n* = 12, 3%) or drug use (*n* = 7, 2%).

### Comparing EZI-PrEP participants across sites

**Fig. 1:**
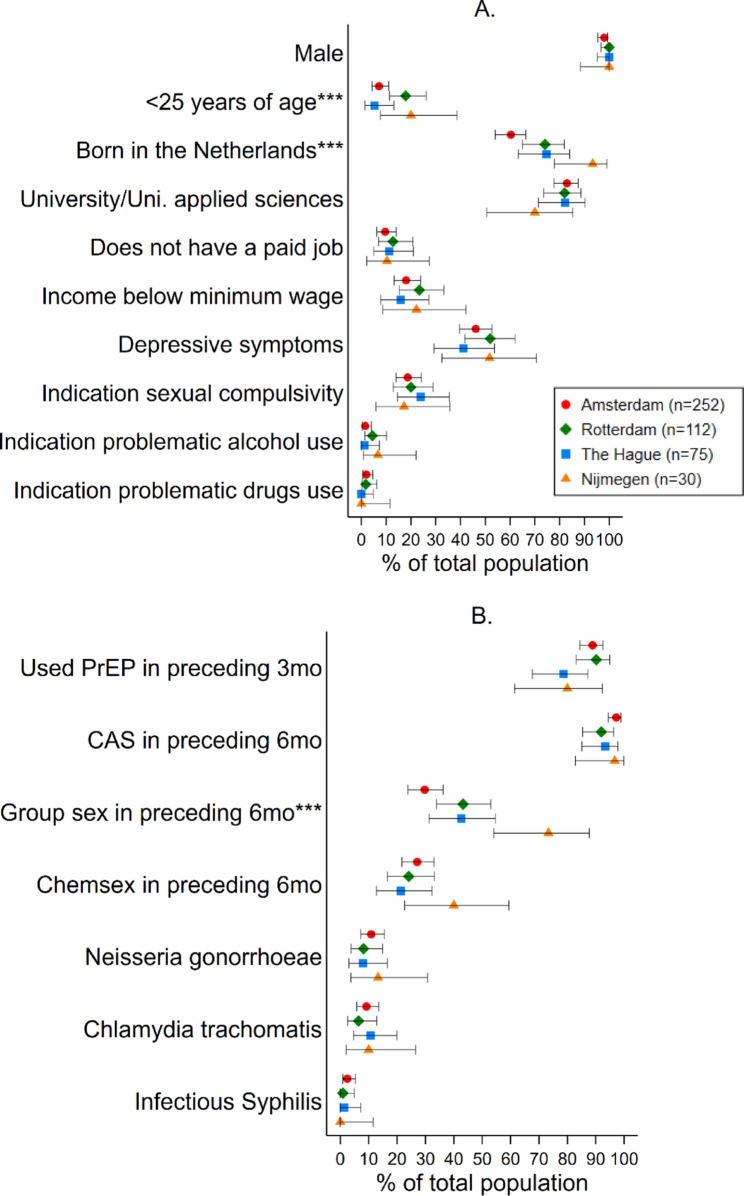
Baseline socio-demographic characteristics, mental health, sexual behaviour and STI prevalence of participants by Centre for Sexual Health, EZI-PrEP study, the Netherlands, September 2021–August 2022. ***p-value < 0.001. Panel **A** shows baseline socio-demographic and mental health characteristics, and Panel **B** shows baseline sexual behaviour and STI prevalence. Percentages are based on complete data; missing/unknown data excluded from calculations. Number of missings can be found in Table 1. Abbreviations: CAS: condomless anal sex; EZI-PrEP: E-Health for Zero Infections - facilitating access to and use of Pre-Exposure Prophylaxis in the Netherlands; PrEP: pre-exposure prophylaxis; STI: sexually transmitted infection; mo: months

252 (54%) participants were included in Amsterdam, 112 (24%) in Rotterdam, 75 (16%) in The Hague, and 30 (6%) in Nijmegen (Table 1). Figure 1 visualizes the distribution of socio-demographic, sexual behaviour and mental health characteristics, as well as the prevalence of bacterial STIs, across these four public health services.

The distribution of age differed between sites. Rotterdam (18%, 20/112) and Nijmegen (20%, 6/30) included a higher proportion of participants between 18 and 25 years of age than Amsterdam (7%, 18/252) and The Hague (5%, 6/75) (*p* = 0.004). Also, the proportion born in the Netherlands was much lower in Amsterdam (60%, 152/252) and higher in Nijmegen (93%, 28/30) compared to Rotterdam (74%, 83/112) and the Hague (75%, 56/75) (*p* < 0.001). Education level did not differ between the sites.

Regarding sexual behaviour in the 6 months before baseline measurement, we found no significant differences between the centres for sexual health in the median number of sex partners, and the percentage of participants reporting insertive anal sex, condomless anal sex, receptive condomless anal sex, chemsex and injecting drugs around or during sex in the 6 months before baseline. However, the proportion of participants who reported receptive anal sex varied substantially between CSHs: 81% (205/252) in Amsterdam, 89% (100/112), in Rotterdam, 93% (70/75) in the Hague, and 83% (25/30) in Nijmegen (*p* = 0.037). The proportion of participants who reported condomless insertive anal sex also varied between CSHs: 88% (223/252) in Amsterdam, 79% (88/112) in Rotterdam, 79% (59/75) in The Hague, and 80% (24/30) in Nijmegen (*p* = 0.042). The percentage of participants who reported group sex was 43% in Rotterdam and the Hague, while higher in Nijmegen (73%, 22/30) and lower in Amsterdam (30%, 66/222), *p* < 0.001, Fig. 1).

### Comparisons of EZI-PrEP participants with other PrEP users

**Fig. 2:**
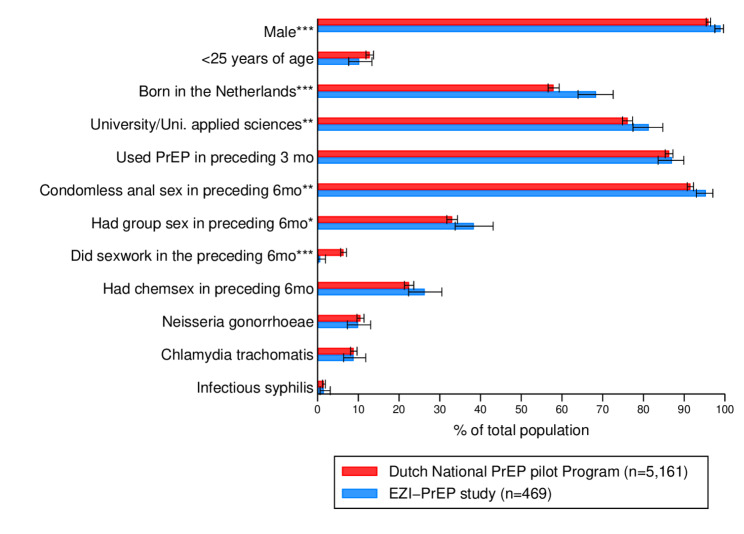
Baseline characteristics and sexual behaviour of EZI-PrEP participants and other PrEP users of the Dutch National PrEP pilot Programme in Centres for Sexual Health in Amsterdam, Rotterdam, The Hague and Nijmegen, September 2021 - August 2022. * p-value < 0.05, ** p-value < 0.01, *** p-value < 0.001. Notes: Percentages are based on complete data; missing/unknown data excluded from calculations. Number of missing can be found in Table 2. Abbreviations: EZI-PrEP: E-Health for Zero Infections - facilitating access to and use of Pre-Exposure Prophylaxis in the Netherlands; PrEP: pre-exposure prophylaxis; mo: months

We included 469 EZI-PrEP participants (464 males and 5 transgender or gender-diverse persons) and 5,161 (4954 males and 207 transgender or gender-diverse persons) other PrEP users of the NPP (Table 2). Figure 2 visualizes the distribution of socio-demographic and sexual behaviour characteristics and STI prevalence of EZI-PrEP and other PrEP users.

EZI-PrEP participants were more often male than the other PrEP users (99% vs. 96% *p* < 0.001), more often born in the Netherlands (68% vs. 58%, *p* < 0.001), and more often completed or were pursuing a college or university degree (82% vs. 77%, *p* = 0.007). EZI-PrEP participants were slightly older (median = 36 years, IQR = 29–47) than other PrEP users (34 years, IQR = 28–44) (*p* = 0.005), but the proportion of individuals below 25 years old was not significantly different (10% and 12% respectively, *p* = 0.12).

With respect to sexual behaviour in the preceding 6 months, we found no significant differences between the two groups in the distribution of the number of sex partners, the percentage participants reporting insertive or receptive anal sex, chemsex and injecting drug use during or around sex in the previous 6 months. However, EZI-PrEP participants more often reported condomless anal sex (95% vs. 92%, *p* = 0.005) and group sex (38% vs. 33%, *p* = 0.024), but less often reported condomless receptive anal sex (83% vs. 88%, *p* = 0.014) and sex work (1% vs. 6%, *p* < 0.001). The overall prevalence of any bacterial STIs was similar in both groups (19% vs. 18%, *p* = 0.799). Also, the prevalence of each individual STI was similar in both groups, i.e., *Chlamydia trachomatis* (9% vs. 9%, *p* = 0.973), *Neisseria gonorrhoea* (10% vs. 11%, *p* = 0.696), infectious syphilis (2% vs. 2%, *p* = 0.737).

**Table 2:** Baseline socio-demographic characteristics, sexual behaviour, STI prevalence and mental health of participants by Centre for Sexual Health, EZI-PrEP study, the Netherlands, September 2021–August 2022

	Amsterdam (*n* = 252)		Rotterdam (*n* = 112)		The Hague (*n* = 75)		Nijmegen (*n* = 30)		*p*-value^2^
	*n* ^1^	%^1^	*n* ^1^	%^1^	*n* ^1^	%^1^	*n* ^1^	%^1^	
**Demographic characteristics**									
**Self-identified gender**									
Male	247	98%	112	100%	75	100%	30	100%	0.446
Transgender or gender-diverse	5	2%	0	0%	0	0%	0	0%	
**Age in years**									
Median, [IQR]	37	[30–47]	32	[27–41]	38	[32–48]	37	[26–53]	< 0.001
< 25 years	18	7%	20	18%	4	5%	6	20%	0.004
25–34 years	84	33%	48	43%	28	37%	8	27%	
35–44 years	68	27%	22	20%	16	21%	5	17%	
≥ 45 years	82	33%	22	20%	27	36%	11	37%	
**Country or region of birth**									
The Netherlands	152	60%	83	74%	56	75%	28	93%	< 0.001^3^
Western and Central Europe and North America	36	14%	9	8%	8	11%	2	7%	
Eastern Europe and Central Asia	14	6%	0	0%	0	0%	0	0%	
Middle East and North Africa	10	4%	1	1%	0	0%	0	0%	
Latin America and the Caribbean	22	9%	9	8%	8	11%	0	0%	
Asia and the Pacific	12	5%	8	7%	2	3%	0	0%	
Sub-Saharan Africa	6	2%	2	2%	1	1%	0	0%	
**Highest completed or** **current education level** ^4^									
None, primary or other	5	2%	0	0%	5	7%	2	7%	0.029
Secondary	37	15%	20	18%	8	11%	7	23%	
College/university	205	83%	91	82%	60	82%	21	70%	
Missing/unknown	5		1		2		0		
** Not having a paid job** ^**5**^	23	10%	13	13%	8	11%	3	10%	0.858
** Income below minimum wage** ^**6**^	39	18%	22	23%	10	16%	6	22%	0.603
**PrEP use past year**									
No use	23	9%	11	10%	14	19%	6	20%	0.076
Used PrEP 4–12 months prior to inclusion	5	2%	0	0%	2	3%	0	0%	
Used PrEP in the 3 months before inclusion	224	89%	101	90%	59	79%	24	80%	
**Sexual behaviour in preceding 6 months**									
** Number of sex partners**,** median [IQR]**^**7**^	7	[4–15]	8	[4–15]	6	[3–10]	9	[5–20]	0.145
** Had insertive anal sex**	227	90%	93	83%	62	83%	24	80%	0.113
** Had receptive anal sex**	205	81%	100	89%	70	93%	25	83%	0.037
** Had condomless anal sex**	245	97%	103	92%	70	93%	29	97%	0.130
** Had condomless insertive anal sex**	223	88%	88	79%	59	79%	24	80%	0.042
** Had condomless receptive anal sex**	202	80%	96	86%	66	88%	24	80%	0.324
** Had group sex** ^**8**^	66	30%	48	43%	32	43%	22	73%	< 0.001
** Did sex work** ^**9**^	0	0%	3	3%	0	0%	0	0%	0.074
** Had chemsex** ^**10**^	68	27%	27	24%	16	21%	12	40%	0.239
** Injected drugs during or around sex** ^**11**^	3	1%	0	0%	0	0%	0	0%	0.793
**Bacterial STI diagnoses at inclusion**									
** Any bacterial STI** ^**12**^	48	20%	16	15%	14	19%	6	20%	0.696
***Neisseria*** ***gonorrhoeae***^*13*^	26	11%	9	8%	6	8%	4	13%	0.732
***Chlamydia*** ***trachomatis***^*14*^	22	9%	7	6%	8	11%	3	10%	0.715
** Infectious syphilis** ^**15**^	6	2%	1	1%	1	1%	0	0%	0.829
Mental well-being									
** Symptoms of depression** ^**16**^	108	46%	52	52%	28	41%	15	52%	0.521
** Indication for sexual compulsivity** ^**17**^	45	19%	21	20%	17	24%	5	17%	0.781
** AUDIT categories (score range)**									
No or low risk alcohol use (0–7)	158	66%	63	61%	54	76%	20	69%	0.362
Hazardous alcohol use (8–15)	69	29%	31	30%	15	21%	6	21%	
Harmful alcohol use (16–19)	8	3%	5	5%	1	1%	1	3%	
Likely dependent on alcohol (≥ 20)	4	2%	5	5%	1	1%	2	7%	
Missing	13		8		4		1		
**DUDIT** **categories** **(score range)**									
No drugs related problems (0–5)	130	54%	65	64%	43	61%	15%	52%	0.502
Harmful drug use (6–24)	104	44%	35	34%	28	39%	14%	48%	
Likely dependent on drugs (≥ 25)	5	2%	2	2%	0	0%	0%	0%	
Missing	13		10		4		1		

Bold values indicate main categories

1. Unless stated otherwise

2. Based on Kruskal-Wallis tests for continuous variables and Pearson’s χ^2^ or Fisher’s exact test for categorical variables

3. P-value is based on binary comparison of individuals born in the Netherlands versus not born in the Netherlands

4.None or Primary or other: no education, elementary school, lbo, mavo, vmbo, mbo-1; Secondary: mbo-2-4, havo, vwo; University or College: university of applied sciences, university. Binary comparison of college/university vs. secondary, none/primary/other: Amsterdam 83% (*n* = 205), Rotterdam 82% (*n* = 91), The Hague 82% (*n* = 60), Nijmegen 70% (*n* = 21), p-value 0.384

5. Included in not having a paid job: student/school (*n* = 23), volunteer (*n* = 3), unemployed (*n* = 10), unable to work (*n* = 4), retired (*n* = 7). Missing/unkown: Amsterdam (*n* = 13), Rotterdam (*n* = 10), The Hague (*n* = 4), Nijmegen (*n* = 1)

6. Minimum wage 2021: € 1701 gross monthly income. Missing/not willing to disclose: Amsterdam (*n* = 36), Rotterdam (*n* = 18), The Hague (*n* = 12), Nijmegen (*n* = 3)

7. Missing/unkown: Amsterdam (*n* = 6), Rotterdam (*n* = 0), The Hague (*n* = 6), Nijmegen (*n* = 0)

8. Missing/unkown: Amsterdam (*n* = 30), Rotterdam (*n* = 1), The Hague (*n* = 0), Nijmegen (*n* = 0)

9. Missing/unkown: Amsterdam (*n* = 7), Rotterdam (*n* = 0), The Hague (*n* = 14), Nijmegen (*n* = 0)

10. Chemsex: sex under influence of one or more of the following drugs: crystal methamphetamine; mephedrone; GHB/GBL; ketamine. Missing/unkown: Amsterdam (*n* = 1), Rotterdam (*n* = 0), The Hague (*n* = 0), Nijmegen (*n* = 0)

11. Drugs included: 3-mmc; heroin; ketamine; cocaine. Missing/unkown: Amsterdam (*n* = 1), Rotterdam (*n* = 0), The Hague (*n* = 0), Nijmegen (*n* = 0)

12. Having at least 1 bacterial STI. Missing/unkown: Amsterdam (*n* = 12), Rotterdam (*n* = 3), The Hague (*n* = 0), Nijmegen (*n* = 0)

13. Missing/unknown: Amsterdam (*n* = 13), Rotterdam (*n* = 2), The Hague (*n* = 0), Nijmegen (*n* = 0)

14. Missing/unknown: Amsterdam (*n* = 13), Rotterdam (*n* = 3), The Hague (*n* = 0), Nijmegen (*n* = 0)

15. Infectious syphilis: primary syphilis, secondary syphilis or early latent syphilis. Missing/unknown: Amsterdam (*n* = 7), Rotterdam (*n* = 2), The Hague (*n* = 0), Nijmegen (*n* = 0)

16. Patient Health Questionnaire-9 (PHQ- 9) score ≥ 5. Missing/unknown: Amsterdam (*n* = 18), Rotterdam (*n* = 12), The Hague (*n* = 7), Nijmegen (*n* = 1)

17. Sexual compulsivity scale (SCS) score ≥ 24. Missing/unknown: Amsterdam (*n* = 11), Rotterdam (*n* = 7), The Hague (*n* = 4), Nijmegen (*n* = 1)

### Comparing EZI-PrEP participants and other PrEP users per centre for sexual health

With respect to socio-demographic variables, we found that EZI-PrEP participants in The Hague and Nijmegen were largely similar to the other PrEP users in the respective centres for sexual health, although the median age of EZI-PrEP participants in The Hague was higher (38 years [IQR 32–48]) than the other PrEP users in The Hague (34 years [IQR 27–46], *p* = 0.012) (Supplementary Tables 1c-d). EZI-PrEP participants in Rotterdam had a similar age to the other PrEP users in Rotterdam and more often completed or were pursuing a college or university degree (82% vs. 72%, *p* = 0.023) (Supplementary Table 1b). EZI-PrEP participants in Amsterdam were older (median = 37 years [IQR 30–47]) than the other PrEP users in Amsterdam (33 years [IQR 28–43], *p* < 0.001) and were more often born in the Netherlands (60% vs. 51%, *p* = 0.013). However, a similar proportion of EZI-PrEP participants and other PrEP users in Amsterdam completed or were pursuing a college or university degree (83% vs. 81%, *p* = 0.354) (Supplementary Table 1a).

With respect to sexual behaviour in the preceding 6 months, we found no significant differences between EZI-PrEP participants and other PrEP users in Rotterdam and The Hague. Overall, EZI-PrEP participants in Nijmegen reported similar sexual behaviour as the other PrEP users in Nijmegen. However, EZI-PrEP participants in Nijmegen reported a higher median number of sex partners (median = 9 [IQR 5–20]) than other PrEP users in Nijmegen (median = 5 [IQR 3–10], *p* = 0.013) and more often reported group sex (73% vs. 50%, *p* = 0.013). For Amsterdam, we found no significant differences between the two populations in the distribution of the number of sex partners, group sex, and the percentage of participants reporting receptive anal sex, condomless receptive anal sex, chemsex and injecting drug use during or around sex, in the past 6 months. However, EZI-PrEP participants more often reported insertive anal sex (90% vs. 85%, *p* = 0.038), condomless anal sex (97% vs. 91%, *p* < 0.001) and condomless insertive anal sex (88% vs. 79%, *p* < 0.001), but less often sex work (0% vs. 10%, *p* < 0.001) than the other PrEP users in Amsterdam. The prevalence of bacterial STIs was similar in both populations across all four centres for sexual health.

## Discussion

In this study, we compared EZI-PrEP participants with other PrEP users in the NPP to assess representativeness of the EZI-PrEP study participants. We observed some differences in characteristics between the two populations, most noteworthy is that EZI-PrEP participants were less often transgender or gender-diverse, slightly older, more often born in the Netherlands, and more often had completed or were pursuing a university or college degree. Furthermore, we found that EZI-PrEP participants reported sexual behaviours associated with STI acquisition more often but reported engaging in sex work less often. However, notwithstanding these differences, we observed that the STI prevalence rates were similar in the two populations.

There were differences in sexual behaviours between EZI-PrEP participants and other PrEP users (i.e., more group sex and condomless anal sex). The numerical differences were nonetheless small and unlikely to be of major clinical relevance. Indeed, STI prevalence did not differ between the groups. When comparing the STI prevalence in the EZI-PrEP and NPP populations in our study to national STI-positivity rates among MSM who received PrEP care at the centres for sexual health in 2022, we found similar rates (i.e., gonorrhoea 9.2%, chlamydia 9.4%, infectious syphilis 1.7%) [37]. This concordance with national data further supports our observation that STI rates among EZI-PrEP participants closely reflect those observed in the broader MSM population using PrEP. In studies of key populations where PrEP is not being used, the presence of an STI is highly associated with HIV acquisition and is therefore considered to be an adequate proxy for the risk of HIV acquisition [38, 39]. Even though EZI-PrEP participants reported condomless sex and group sex more often than those in the NPP, the prevalence of STIs between populations was comparable. As such, the risk of acquiring HIV among EZI-PrEP participants can be considered comparable to that of the broader population of PrEP users who receive publicly funded PrEP care in the Netherlands.

Certain groups were clearly underrepresented in the EZI-PrEP study. Transgender or gender-diverse individuals comprised only 1% of EZI-PrEP participants compared to 4% of the other PrEP users in the NPP. Furthermore, PrEP users who were not born in the Netherlands were less commonly enrolled in the EZI-PrEP trial. Prior research has suggested that transgender and gender-diverse persons, as well as recently migrated MSM, face specific barriers to HIV and PrEP care and a higher risk of HIV acquisition [40–44]. Individuals who completed no or primary schooling were less often enrolled in the EZI-PrEP trial compared to other PrEP users. While the level of education may not necessarily directly influence the risk of HIV acquisition, it can affect the effective use of telehealth services, potentially increasing HIV acquisition risk due to lower technological or medical literacy [45–48]. Finally, engaging in sex work was less commonly reported among EZI-PrEP participants, while individuals engaging in sex work are known to belong to a key population at a higher risk of HIV acquisition [49–52]. Taken together, some caution is warranted when extrapolating the findings from the EZI-PrEP trial to these underrepresented groups. Each of these groups may have varying preferences and needs regarding the frequency and modality of PrEP monitoring. While some individuals may benefit from less frequent or remote monitoring (e.g. people who may wish to demedicalize aspects of their life), others may prefer regular in-clinic visits, for example to facilitate HIV and STI testing (e.g. individuals engaged in sex work). Tailored PrEP care will thus be essential to meet the specific preferences and needs of these populations. Should less frequent or online monitoring be implemented in routine care, these PrEP care models could offer greater flexibility for tailored PrEP care and free up resources to support individuals who require more intensive PrEP care.

Within the EZI-PrEP population, comparisons across sites revealed that participants at the four study sites were broadly comparable. Notable differences included the younger age of participants in Rotterdam and a higher percentage of participants who reported group sex in Nijmegen. In parallel, when comparing the EZI-PrEP participants in Rotterdam and Nijmegen to their respective populations of other PrEP users in the NPP, we observed the same differences in socio-demographics and sexual behaviour. These differences may suggest a selection effect, with certain groups being over- or underrepresented due to site-specific factors. Given the small sample size in Nijmegen (*n* = 30), such selection effects could be more pronounced, potentially limiting the generalizability of the findings from this site. However, considering that the STI prevalence was similar between EZI-PrEP participants and other PrEP users within centres, these differences in socio-demographics or sexual behaviours are unlikely to have a clinically meaningful effect.

There are several limitations worth mentioning. First, certain groups were less likely to be included in the EZI-PrEP trial due to the study procedures and eligibility criteria. Participation in the EZI-PrEP trial required access to a smartphone, postal address, internet connection, and the ability to complete online bank transactions, which may have limited participation PrEP users who are economically or socially vulnerable, such as persons experiencing homelessness and those who are undocumented, or who have limited digital skills. This limitation was unavoidable due to the online-mediated PrEP monitoring arms. In addition, cisgender women and individuals under 18 years of age were not eligible for the EZI-PrEP trial. Second, the EZI-PrEP study was conducted in urban centres for sexual health and therefore did not include PrEP users living in more rural areas, where longer travel distances or transportation challenges have been reported as barriers to PrEP care [10, 53]. Third, we did not compare EZI-PrEP participants with individuals receiving PrEP through their general practitioner, which may be a distinct group of PrEP users. For each of these populations, caution is warranted when extrapolating the findings of the EZI-PrEP trial, as their preferences and needs regarding PrEP monitoring may differ. Fourth, given the large sample of other PrEP users (*n* = 5,161), even small and clinically irrelevant differences may have reached statistical significance.

In conclusion, the characteristics of EZI-PrEP participants are largely comparable to those of other PrEP users in the NPP, and hence the results of the EZI-PrEP trial could be considered broadly generalizable to the wider population of PrEP users receiving publicly funded PrEP care in the Netherlands. Nevertheless, the underrepresentation of transgender and gender-diverse persons, individuals born outside the Netherlands, sex workers and people with no or primary education may limit the generalizability of the trial findings to these groups. Future research should therefore focus on developing and evaluating PrEP care strategies that address the needs of these underrepresented populations.

## Electronic Supplementary Material

Below is the link to the electronic supplementary material.

Supplementary Material 1.

## Data Availability

The EZI-PrEP data are owned by the EZI-PrEP consortium. EZI-PrEP data can be requested by submitting a study proposal to the EZI-PrEP steering committee. It will be possible to obtain a proposal format from the Public Health Services of Amsterdam via datamanagersoz@ggd.amsterdam.nl. Requests for further information can also be submitted through the same email addresses. The EZI-PrEP steering committee will verify each proposal for compatibility with general objectives, ethical approval, and informed consent forms of the EZI-PrEP trial and potential overlap with ongoing studies. There will be no restrictions to obtaining the data and all data requests will be processed in a similar way.

## Abbreviations

AUDIT: Alcohol use disorders identification test
CSH: Centre for sexual health
DUDIT: Drug use disorders identification test
EZI-PrEP: E-health for zero infections–facilitating access to and use of pre-exposure prophylaxis in the Netherlands
GBL: Gamma-butyrolactone
GHB: Gamma-hydroxybutyrate
HIV: Human immunodeficiency virus
IQR: Interquartile range
MSM: Men who have sex with men
NPP: National PrEP pilot programme
PEP: Post-exposure prophylaxis
PHQ-9: Patient health questionnaire-9
PrEP: Pre-exposure prophylaxis
RCT: Randomized controlled trial
RIVM: National Institute for Public Health and the Environment
SCS: Sexual Compulsivity Scale
STI: Sexually transmitted infection
TGDP: Transgender or gender-diverse persons
