# Supplementary material for: Representativeness of participants in a randomized controlled trial on modalities of monitoring oral HIV pre-exposure prophylaxis use
Source: AIDS Res Ther. 2026 Apr 13;23:68. doi: 10.1186/s12981-026-00886-1 (PMC13362004; doi:10.1186/s12981-026-00886-1)
Supplement: Supplementary file 1 — Supplementary Material 1. [file 12981_2026_886_MOESM1_ESM.docx]

**Supplement for manuscript entitled:**

*Representativeness of participants in a randomized controlled trial on modalities of monitoring oral HIV pre-exposure prophylaxis use*

Marije L. GROOT BRUINDERINK^1,2^, Maarten F. SCHIM VAN DER LOEFF^1,3,4,5^, Florien DUSSELDORP^1^, Katja VAN DER VELDE^1^, Laura BLITZ^6^, Jean-Marie BRAND^6^, Colette C. A. J. VAN BOKHOVEN^7^, Joey WOUDSTRA^1^, Koenraad VERMEY^8^, Sophie BOERS^9^, Maudy SLUIMER^9^, Hannelore M. GÖTZ^9^, Anders BOYD^1,3,4,10^, Lotte WERNER^1^, Maaike L. SOORS d’ANCONA^11,12,13^, Frenk VAN HARREVELD^2^, Maria PRINS^1,3,4,5^, Elske HOORNENBORG^1,3,4,5^, Udi DAVIDOVICH^1,2*^ , Vita W. JONGEN^1,4,10*^

** These authors contributed equally*

Institutional affiliations:

1. Department of Infectious Diseases, Public Health Service Amsterdam, Amsterdam, The Netherlands
2. University of Amsterdam, Department of Psychology, Amsterdam, The Netherlands
3. Amsterdam UMC location University of Amsterdam, Department of Internal Medicine, Meibergdreef 9, Amsterdam, The Netherlands
4. Amsterdam Institute for Immunology & Infectious Diseases (AII), Amsterdam, the Netherlands
5. Amsterdam Public Health research institute (APH), Amsterdam, the Netherlands
6. Public Health Service of Haaglanden, Department of Sexual Health, The Hague, The Netherlands
7. Public Health Service of Gelderland-Zuid, Department of Sexual Health, Nijmegen, The Netherlands
8. STI AIDS, The Netherlands
9. Public Health Service of Rotterdam-Rijnmond, Department of Infectious Diseases, Rotterdam, The Netherlands
10. Stichting hiv monitoring, Amsterdam, the Netherlands
11. Amsterdam UMC location University of Amsterdam, Public and Occupational Health, Meibergdreef 9, Amsterdam, The Netherlands
12. Amsterdam Public Health research institute, Quality of Care, Amsterdam, The Netherlands
13. Amsterdam UMC, Centre for Sustainable Healthcare, Amsterdam, The Netherlands

**Supplementary Table 1a:** Baseline socio-demographic characteristics and sexual behaviour of EZI-PrEP participants and other PrEP users of the Dutch National PrEP pilot Programme at the Centre for Sexual Health in Amsterdam, September 2021 - August 2022

**Supplementary Table 1b:** Baseline socio-demographic characteristics and sexual behaviour of EZI-PrEP participants and other PrEP users of the Dutch National PrEP pilot Programme at the Centre for Sexual Health in Rotterdam, September 2021 - August 2022

**Supplementary Table 1c:** Baseline socio-demographic characteristics and sexual behaviour of EZI-PrEP participants and other PrEP users of the Dutch National PrEP pilot Programme at the Centre for Sexual Health in the Hague, September 2021 - August 2022

**Supplementary Table 1d:** Baseline socio-demographic characteristics and sexual behaviour of EZI-PrEP participants and other PrEP users of the Dutch National PrEP pilot Programme at the Centre for Sexual Health in Nijmegen, September 2021 - August 2022

| **Supplementary Table 1a: Baseline socio-demographic characteristics and sexual behaviour of EZI-PrEP participants and other PrEP users of the Dutch National PrEP pilot Programme at the**  **Centre for Sexual Health in Amsterdam, September 2021 - August 2022** | | | | | |
| --- | --- | --- | --- | --- | --- |
|  | **EZI-PrEP**  **Amsterdam**  **(n=252)** | | **NPP**  **Amsterdam**  **(n=3026)** | |  |
|  | *n^1^* | *%^1^* | *n^1^* | *%^1^* | *p-value^2^* |
| **Demographic characteristics** |  |  |  |  |  |
| **Gender** |  |  |  |  |  |
| Male | 247 | 98% | 2,861 | 95% | 0.017 |
| Transgender or gender diverse | 5 | 2% | 165 | 5% |  |
| **Age in years** |  |  |  |  |  |
| Median, [IQR] | 37 | [30-47] | 33 | [28-43] | <0.001 |
| <25years^3^ | 18 | 7% | 378 | 12% | <0.001 |
| 25-34 years | 84 | 33% | 1,238 | 41% |  |
| 35-44 years | 68 | 27% | 733 | 24% |  |
| ≥45 years | 82 | 33% | 677 | 22% |  |
| **Country of birth** |  |  |  |  |  |
| The Netherlands^4^ | 152 | 60% | 1,534 | 51% | 0.013 |
| Western and Central Europe and North America | 36 | 14% | 410 | 14% |  |
| Eastern Europe and Central Asia | 14 | 6% | 175 | 6% |  |
| Middle East and North Africa | 10 | 4% | 275 | 9% |  |
| Latin America and the Caribbean | 22 | 9% | 394 | 13% |  |
| Asia and the Pacific | 12 | 5% | 181 | 6% |  |
| Sub-Saharan Africa | 6 | 2% | 47 | 2% |  |
| Missing / unknown | 0 |  | 10 |  |  |
| **Highest completed or current education level^5^** | | | | | |
| None, primary or other | 5 | 2% | 189 | 7% | 0.005 |
| Secondary | 37 | 15% | 321 | 12% |  |
| College/university | 205 | 83% | 2,114 | 81% |  |
| Missing / unknown | 5 |  | 402 |  |  |
| **PrEP use past year** |  |  |  |  |  |
| No use | 23 | 9% | 314 | 11% | 0.656 |
| Used PrEP 4-12 months prior to inclusion | 5 | 2% | 74 | 3% |  |
| Used PrEP in the 3 months before inclusion | 224 | 89% | 2,572 | 87% |  |
| Unknown/missing | 0 |  | 66 |  |  |
| **Sexual behaviour in preceding 6 months** |  |  |  |  |  |
| **Number of sex partners, median [IQR]^6^** | 7 | [4-15] | 6 | [3-15] | 0.164 |
| **Had insertive anal sex** | 227 | 90% | 2,582 | 85% | 0.038 |
| **Had receptive anal sex** | 205 | 81% | 2,479 | 82% | 0.820 |
| **Had condomless anal sex** | 245 | 97% | 2,743 | 91% | <0.001 |
| **Had condomless insertive anal sex** | 223 | 88% | 2,380 | 79% | <0.001 |
| **Had condomless receptive anal sex** | 202 | 80% | 2,292 | 76% | 0.114 |
| **Had group sex^7^** | 66 | 30% | 791 | 27% | 0.335 |
| **Did sex work^8^** | 0 | 0% | 241 | 10% | <0.001 |
| **Had chemsex^9^** | 68 | 27% | 699 | 23% | 0.160 |
| **Injected drugs during or around sex^10^** | 3 | 1% | 32 | 1% | 0.748 |
| **Bacterial STI diagnoses** |  |  |  |  |  |
| **Any bacterial STI^11^** | 48 | 20% | 556 | 19% | 0.741 |
| ***Neisseria gonorrhoeae^12^*** | 26 | 11% | 344 | 12% | 0.661 |
| ***Chlamydia trachomatis^13^*** | 22 | 9% | 268 | 9% | 0.994 |
| **Infectious syphilis^14^** | 6 | 2% | 47 | 2% | 0.303 |
| *1. Unless stated otherwise*  *2. Based on Wilcoxon rank-sum tests for continuous variables and Pearson’s χ^2^ or Fisher’s exact for categorical variables.*  *3. Binary comparison of 18-25 year old vs ≥25 years old, p-value 0.012.*  *4. Binary comparison of those born in the Netherlands vs not born in the Netherlands, p-value 0.004.*  *5. None or Primary or other: no education, elementary school, lbo, mavo, vmbo, mbo-1; Secondary: mbo-2-4, havo, vwo; University or College: university of applied sciences, university. Binary comparison of college/university vs. secondary, none/primary/other, p-value* *0.354.*  *6. Missing/unkown: EZI-PrEP (n=6), NPP (n=66).*  *7. Missing/unkown: EZI-PrEP (n=30), NPP (n=69).*  *8. Missing/unkown: EZI-PrEP (n=7), NPP (n=545).*  *9. Chemsex: sex under influence of one or more of the following drugs: crystal methamphetamine; mephedrone; GHB/GBL; ketamine. Missing/unkown: EZI-PrEP (n=1),NPP (n=10).*  *10. Drugs included: 3-mmc; heroin; ketamine; cocaine. Missing/unkown: EZI-PrEP (n=1),NPP (n=10).*  *11. Having at least 1 bacterial STI. Missing/unknown: EZI-PrEP (n=12),NPP (n=119).*  *12. Missing/unknown: EZI-PrEP (n=13),NPP (n=118).*  *13. Missing/unknown: EZI-PrEP (n=13),NPP (n=119).*  *14. Infectious syphilis: Lues I, Lues II, Lues latens recens. Missing/unkown: EZI-PrEP (n=7),NPP (n=49).*  *Note: Proportions are based on complete data; missing/unknown data excluded from calculations.*  *EZI-PrEP: E-Health for Zero Infections - facilitating access to and use of Pre-Exposure Prophylaxis in the Netherlands; IQR: interquartile range; PrEP: pre-exposure prophylaxis; STI: sexually transmitted infection.* | | | | | |

| **Supplementary Table 1b: Baseline socio-demographic characteristics and sexual behaviour of EZI-PrEP participants and other PrEP users of the Dutch National PrEP pilot Programme at the**  **Centre for Sexual Health in Rotterdam, September 2021 - August 2022** | | | | | |
| --- | --- | --- | --- | --- | --- |
|  | **EZI-PrEP**  **Rotterdam**  **(n=112)** | | **NPP**  **Rotterdam**  **(n=1233)** | |  |
|  | *n^1^* | *%^1^* | *n^1^* | *%^1^* | *p-value^2^* |
| **Demographic characteristics** |  |  |  |  |  |
| **Gender** |  |  |  |  |  |
| Male | 112 | 100% | 1,216 | 99% | 0.389 |
| Transgender or gender diverse | 0 | 0% | 17 | 1% |  |
| **Age in years** |  |  |  |  |  |
| Median, [IQR] | 32 | 27-41 | 35 | 28-43 | 0.058 |
| <25years^3^ | 20 | 18% | 150 | 12% | 0.098 |
| 25-34 years | 48 | 43% | 460 | 37% |  |
| 35-44 years | 22 | 20% | 336 | 27% |  |
| ≥45 years | 22 | 20% | 287 | 23% |  |
| **Country of birth** |  |  |  |  |  |
| The Netherlands^4^ | 83 | 74% | 806 | 65% | 0.011 |
| Western and Central Europe and North America | 9 | 8% | 80 | 6% |  |
| Eastern Europe and Central Asia | 0 | 0% | 54 | 4% |  |
| Middle East and North Africa | 1 | 1% | 60 | 5% |  |
| Latin America and the Caribbean | 9 | 8% | 159 | 13% |  |
| Asia and the Pacific | 8 | 7% | 51 | 4% |  |
| Sub-Saharan Africa | 2 | 2% | 22 | 2% |  |
| Missing / unknown | 0 |  | 1 |  |  |
| **Highest completed or current education level^5^** | | | | | |
| None, primary or other | 0 | 0% | 49 | 4% | 0.013 |
| Secondary | 20 | 18% | 289 | 24% |  |
| College/university | 91 | 82% | 868 | 72% |  |
| Missing / unknown | 1 |  | 27 |  |  |
| **PrEP use past year** |  |  |  |  |  |
| No use | 11 | 10% | 63 | 5% | 0.067 |
| Used PrEP 4-12 months prior to inclusion | 0 | 0% | 18 | 1% |  |
| Used PrEP in the 3 months before inclusion | 101 | 90% | 1,149 | 93% |  |
| Unknown/missing | 0 |  | 3 |  |  |
| **Sexual behaviour in preceding 6 months** |  |  |  |  |  |
| **Number of sex partners, median [IQR]^6^** | 8 | [4-15] | 6 | [4-12] | 0.278 |
| **Had insertive anal sex** | 93 | 83% | 1,066 | 86% | 0.315 |
| **Had receptive anal sex** | 100 | 89% | 1,059 | 86% | 0.319 |
| **Had condomless anal sex** | 103 | 92% | 1,158 | 94% | 0.413 |
| **Had condomless insertive anal sex** | 88 | 79% | 1,005 | 82% | 0.446 |
| **Had condomless receptive anal sex** | 96 | 86% | 986 | 80% | 0.142 |
| **Had group sex^7^** | 48 | 43% | 525 | 43% | 0.971 |
| **Did sex work^8^** | 3 | 3% | 24 | 2% | 0.488 |
| **Had chemsex^9^** | 27 | 24% | 269 | 22% | 0.582 |
| **Injected drugs during or around sex^10^** | 0 | 0% | 8 | 1% | 1.000 |
| **Bacterial STI diagnoses** |  |  |  |  |  |
| **Any bacterial STI^11^** | 16 | 15% | 194 | 16% | 0.685 |
| ***Neisseria gonorrhoeae^12^*** | 9 | 8% | 108 | 9% | 0.785 |
| ***Chlamydia trachomatis^13^*** | 7 | 6% | 97 | 8% | 0.547 |
| **Infectious syphilis^14^** | 1 | 1% | 15 | 1% | 1.000 |
| *1. Unless stated otherwise*  *2. Based on Wilcoxon rank-sum tests for continuous variables and Pearson’s χ2 or Fisher’s exact for categorical variables.*  *3. Binary comparison of 18-25 year old vs ≥25 years old, p-value 0.083.*  *4. Binary comparison of those born in the Netherlands vs not born in the Netherlands, p-value 0.063.*  *5. None or Primary or other: no education, elementary school, lbo, mavo, vmbo, mbo-1; Secondary: mbo-2-4, havo, vwo; University or College: university of applied sciences, university. Binary comparison of college/university vs. secondary, none/primary/other, p-value* *0.023.*  *6. Missing/unkown: EZI-PrEP (n=0), NPP (n=1).*  *7. Missing/unkown: EZI-PrEP (n=1), NPP (n=24).*  *8. Missing/unkown: EZI-PrEP (n=0), NPP (n=3).*  *9. Chemsex: sex under influence of one or more of the following drugs: crystal methamphetamine; mephedrone; GHB/GBL; ketamine. Missing/unkown: EZI-PrEP (n=0),NPP (n=2).*  *10. Drugs included: 3-mmc; heroin; ketamine; cocaine. Missing/unkown: EZI-PrEP (n=0),NPP (n=2).*  *11. Having at least 1 bacterial STI. Missing/unknown: EZI-PrEP (n=3),NPP (n=33).*  *12. Missing/unknown: EZI-PrEP (n=2),NPP (n=27).*  *13. Missing/unknown: EZI-PrEP (n=3),NPP (n=28).*  *14. Infectious syphilis: Lues I, Lues II, Lues latens recens. Missing/unkown: EZI-PrEP (n=2),NPP (n=16).*  *Note: Proportions are based on complete data; missing/unknown data excluded from calculations.*  *EZI-PrEP: E-Health for Zero Infections - facilitating access to and use of Pre-Exposure Prophylaxis in the Netherlands; IQR: interquartile range; PrEP: pre-exposure prophylaxis; STI: sexually transmitted infection.* | | | | | |

| **Supplementary Table 1c: Baseline socio-demographic characteristics and sexual behaviour of EZI-PrEP participants and other PrEP users of the Dutch National PrEP pilot Programme at the**  **Centre for Sexual Health in the Hague, September 2021 - August 2022** | | | | | |
| --- | --- | --- | --- | --- | --- |
|  | **EZI-PrEP**  **The Hague**  **(n=75)** | | **NPP**  **The Hague**  **(n=542)** | |  |
|  | *n^1^* | *%^1^* | *n^1^* | *%^1^* | *p-value^2^* |
| **Demographic characteristics** |  |  |  |  |  |
| **Gender** |  |  |  |  |  |
| Male | 75 | 100% | 520 | 96% | 0.095 |
| Transgender or gender diverse | 0 | 0% | 22 | 4% |  |
| **Age in years** |  |  |  |  |  |
| Median, [IQR] | 38 | [32-48] | 34 | [27-46] | 0.012 |
| <25years^3^ | 4 | 5% | 87 | 16% | 0.079 |
| 25-34 years | 28 | 37% | 199 | 37% |  |
| 35-44 years | 16 | 21% | 105 | 19% |  |
| ≥45 years | 27 | 36% | 151 | 28% |  |
| **Country of birth** |  |  |  |  |  |
| The Netherlands^4^ | 56 | 75% | 328 | 61% | 0.050 |
| Western and Central Europe and North America | 8 | 11% | 49 | 9% |  |
| Eastern Europe and Central Asia | 0 | 0% | 24 | 4% |  |
| Middle East and North Africa | 0 | 0% | 27 | 5% |  |
| Latin America and the Caribbean | 8 | 11% | 64 | 12% |  |
| Asia and the Pacific | 2 | 3% | 39 | 7% |  |
| Sub-Saharan Africa | 1 | 1% | 9 | 2% |  |
| Missing / unknown | 0 |  | 2 |  |  |
| **Highest completed or current education level^5^** |  |  |  |  |  |
| None, primary or other | 5 | 7% | 48 | 10% | 0.526 |
| Secondary | 8 | 11% | 70 | 14% |  |
| College/university | 60 | 82% | 379 | 76% |  |
| Missing / unknown | 2 |  | 45 |  |  |
| **PrEP use past year** |  |  |  |  |  |
| No use | 14 | 19% | 163 | 31% | 0.075 |
| Used PrEP 4-12 months prior to inclusion | 2 | 3% | 16 | 3% |  |
| Used PrEP in the 3 months before inclusion | 59 | 79% | 353 | 66% |  |
| Unknown/missing | 0 |  | 10 |  |  |
| **Sexual behaviour in preceding 6 months** |  |  |  |  |  |
| **Number of sex partners, median [IQR]^6^** | 6 | [3-10] | 6 | [4-12] | 0.483 |
| **Had insertive anal sex** | 62 | 83% | 451 | 83% | 0.906 |
| **Had receptive anal sex** | 70 | 93% | 486 | 90% | 0.319 |
| **Had condomless anal sex** | 70 | 93% | 497 | 92% | 0.627 |
| **Had condomless insertive anal sex** | 59 | 79% | 421 | 78% | 0.846 |
| **Had condomless receptive anal sex** | 66 | 88% | 452 | 83% | 0.308 |
| **Had group sex^7^** | 32 | 43% | 179 | 33% | 0.109 |
| **Did sex work^8^** | 0 | 0% | 22 | 4% | 0.153 |
| **Had chemsex^9^** | 16 | 21% | 81 | 15% | 0.154 |
| **Injected drugs during or around sex^10^** | 0 | 0% | 7 | 1% | 1.000 |
| **Bacterial STI diagnoses** |  |  |  |  |  |
| **Any bacterial STI^11^** | 14 | 19% | 84 | 16% | 0.583 |
| ***Neisseria gonorrhoeae^12^*** | 6 | 8% | 46 | 9% | 0.808 |
| ***Chlamydia trachomatis^13^*** | 8 | 11% | 48 | 9% | 0.691 |
| **Infectious syphilis^14^** | 1 | 1% | 7 | 1% | 1.000 |
| *1. Unless stated otherwise*  *2. Based on Wilcoxon rank-sum tests for continuous variables and Pearson’s χ2 or Fisher’s exact for categorical variables.*  *3. Binary comparison of 18-25 year old vs ≥25 years old, p-value 0.014.*  *4. Binary comparison of those born in the Netherlands vs not born in the Netherlands, p-value 0.020.*  *5. None or Primary or other: no education, elementary school, lbo, mavo, vmbo, mbo-1; Secondary: mbo-2-4, havo, vwo; University or College: university of applied sciences, university. Binary comparison of college/university vs. secondary, none/primary/other, p-value* *0.260.*  *6. Missing/unkown: EZI-PrEP (n=6), NPP (n=26).*  *7. Missing/unkown: EZI-PrEP (n=0), NPP (n=4).*  *8. Missing/unkown: EZI-PrEP (n=14), NPP (n=26).*  *9. Chemsex: sex under influence of one or more of the following drugs: crystal methamphetamine; mephedrone; GHB/GBL; ketamine. Missing/unkown: EZI-PrEP (n=0),NPP (n=0).*  *10. Drugs included: 3-mmc; heroin; ketamine; cocaine. Missing/unkown: EZI-PrEP (n=0),NPP (n=0).*  *11. Having at least 1 bacterial STI. Missing/unknown: EZI-PrEP (n=0),NPP (n=22).*  *12. Missing/unknown: EZI-PrEP (n=0),NPP (n=22).*  *13. Missing/unknown: EZI-PrEP (n=0),NPP (n=22).*  *14. Infectious syphilis: Lues I, Lues II, Lues latens recens. Missing/unkown: EZI-PrEP (n=0),NPP (n=19).*  *Note: Proportions are based on complete data; missing/unknown data excluded from calculations.*  *EZI-PrEP: E-Health for Zero Infections - facilitating access to and use of Pre-Exposure Prophylaxis in the Netherlands; IQR: interquartile range; PrEP: pre-exposure prophylaxis; STI: sexually transmitted infection.* | | | | | |

| **Supplementary Table 1d: Baseline socio-demographic characteristics and sexual behaviour of EZI-PrEP participants and other PrEP users of the Dutch National PrEP pilot Programme at the**  **Centre for Sexual Health in Nijmegen, September 2021 - August 2022** | | | | | |
| --- | --- | --- | --- | --- | --- |
|  | **EZI-PrEP**  **Nijmegen**  **(n=30)** | | **NPP**  **Nijmegen**  **(n=360)** | |  |
|  | *n^1^* | *%^1^* | *n^1^* | *%^1^* | *p-value^2^* |
| **Demographic characteristics** |  |  |  |  |  |
| **Gender** |  |  |  |  |  |
| Male | 30 | 100% | 357 | 99% | 0.616 |
| Transgender or gender diverse | 0 | 0% | 3 | 1% |  |
| **Age in years** |  |  |  |  |  |
| Median, [IQR] | 37 | [26-53] | 43 | [31-53] | 0.169 |
| <25years^3^ | 6 | 20% | 40 | 11% | 0.394 |
| 25-34 years | 8 | 27% | 78 | 22% |  |
| 35-44 years | 5 | 17% | 79 | 22% |  |
| ≥45 years | 11 | 37% | 163 | 45% |  |
| **Country of birth** |  |  |  |  |  |
| The Netherlands^4^ | 28 | 93% | 314 | 87% | 0.782 |
| Western and Central Europe and North America | 2 | 7% | 9 | 3% |  |
| Eastern Europe and Central Asia | 0 | 0% | 10 | 3% |  |
| Middle East and North Africa | 0 | 0% | 8 | 2% |  |
| Latin America and the Caribbean | 0 | 0% | 10 | 3% |  |
| Asia and the Pacific | 0 | 0% | 4 | 1% |  |
| Sub-Saharan Africa | 0 | 0% | 5 | 1% |  |
| Missing / unknown | 0 |  | 0 |  |  |
| **Highest completed or current education level^5^** | | | | | |
| None, primary or other | 2 | 7% | 35 | 10% | 0.510 |
| Secondary | 7 | 23% | 111 | 32% |  |
| College/university | 21 | 70% | 201 | 58% |  |
| Missing / unknown | 0 |  | 13 |  |  |
| **PrEP use past year** |  |  |  |  |  |
| No use | 6 | 20% | 25 | 7% | 0.023 |
| Used PrEP 4-12 months prior to inclusion | 0 | 0% | 18 | 5% |  |
| Used PrEP in the 3 months before inclusion | 24 | 80% | 315 | 88% |  |
| Unknown/missing | 0 |  | 2 |  |  |
| **Sexual behaviour in preceding 6 months** |  |  |  |  |  |
| **Number of sex partners, median [IQR]^6^** | 9 | [5-20] | 5 | [3-10] | 0.013 |
| **Had insertive anal sex** | 24 | 80% | 305 | 85% | 0.494 |
| **Had receptive anal sex** | 25 | 83% | 305 | 85% | 0.839 |
| **Had condomless anal sex** | 29 | 97% | 328 | 91% | 0.294 |
| **Had condomless insertive anal sex** | 24 | 80% | 286 | 79% | 0.942 |
| **Had condomless receptive anal sex** | 24 | 80% | 288 | 80% | 1.000 |
| **Had group sex^7^** | 22 | 73% | 177 | 50% | 0.013 |
| **Did sex work^8^** | 0 | 0% | 4 | 1% | 0.725 |
| **Had chemsex^9^** | 12 | 40% | 108 | 30% | 0.259 |
| **Injected drugs during or around sex^10^** | 0 | 0% | 8 | 2% | 0.523 |
| **Bacterial STI diagnoses** |  |  |  |  |  |
| **Any bacterial STI^11^** | 6 | 20% | 60 | 17% | 0.660 |
| ***Neisseria gonorrhoeae^12^*** | 4 | 13% | 26 | 7% | 0.273 |
| ***Chlamydia trachomatis^13^*** | 3 | 10% | 30 | 8% | 0.732 |
| **Infectious syphilis^14^** | 0 | 0% | 9 | 3% | 1.000 |
| *1. Unless stated otherwise*  *2. Based on Wilcoxon rank-sum tests for continuous variables and Pearson’s χ2 or Fisher’s exact for categorical variables.*  *3. Binary comparison of 18-25 year old vs ≥25 years old, p-value 0.147.*  *4. Binary comparison of those born in the Netherlands vs not born in the Netherlands, p-value 0.328.*  *5. None or Primary or other: no education, elementary school, lbo, mavo, vmbo, mbo-1; Secondary: mbo-2-4, havo, vwo; University or College: university of applied sciences, university. Binary comparison of college/university vs. secondary, none/primary/other, p-value* *0.197.*  *6. Missing/unkown: EZI-PrEP (n=0), NPP (n=3).*  *7. Missing/unkown: EZI-PrEP (n=0), NPP (n=5).*  *8. Missing/unkown: EZI-PrEP (n=0), NPP (n=0).*  *9. Chemsex: sex under influence of one or more of the following drugs: crystal methamphetamine; mephedrone; GHB/GBL; ketamine. Missing/unkown: EZI-PrEP (n=0),NPP (n=1).*  *10. Drugs included: 3-mmc; heroin; ketamine; cocaine. Missing/unkown: EZI-PrEP (n=0),NPP (n=1).*  *11. Having at least 1 bacterial STI. Missing/unknown: EZI-PrEP (n=0),NPP (n=4).*  *12. Missing/unknown: EZI-PrEP (n=0),NPP (n=2).*  *13. Missing/unknown: EZI-PrEP (n=0),NPP (n=2).*  *14. Infectious syphilis: Lues I, Lues II, Lues latens recens. Missing/unkown: EZI-PrEP (n=0),NPP (n=3).*  *Note: Proportions are based on complete data; missing/unknown data excluded from calculations.*  *EZI-PrEP: E-Health for Zero Infections - facilitating access to and use of Pre-Exposure Prophylaxis in the Netherlands; IQR: interquartile range; PrEP: pre-exposure prophylaxis; STI: sexually transmitted infection.* | | | | | |
